# Identification of Key Modules, Hub Genes, and Noncoding RNAs in Chronic Rhinosinusitis with Nasal Polyps by Weighted Gene Coexpression Network Analysis

**DOI:** 10.1155/2020/6140728

**Published:** 2020-01-23

**Authors:** Xuanchen Zhou, Xiaoyue Zhen, Yiqing Liu, Zhaoyang Cui, Zhiyong Yue, Anting Xu, Jie Han

**Affiliations:** ^1^Department of Otorhinolaryngology Head and Neck Surgery, Shandong Provincial Hospital Affiliated to Shandong University, Jinan 250021, China; ^2^Minimally Invasive Urology Center, Shandong Provincial Hospital Affiliated to Shandong University, Jinan 250021, China; ^3^Department of Otorhinolaryngology and Head and Neck Surgery, The Second Hospital of Shandong University, Jinan 250033, China

## Abstract

Chronic rhinosinusitis with nasal polyps (CRSwNP) is a chronic inflammatory disease with relatively easy recurrence. However, the precise molecular mechanisms of this disease are poorly known. Based on gene sequencing data obtained from the Gene Expression Omnibus (GEO) database, we constructed coexpression networks by weighted gene coexpression network analysis (WGCNA). Gene Ontology (GO) and Kyoto Encyclopedia of Genes and Genomes (KEGG) enrichment analyses were performed by the Database for Annotation, Visualization, and Integrated Discovery (DAVID). The core gene of pathogenesis, CRSwNP, was screened by protein-protein interaction data (PPI) from the HPRD database. Unsupervised clustering was applied to screen hub genes related to the phenotype of CRSwNP. Blue and turquoise modules were found to be most significantly related to the pathogenicity of CRSwNP. Functional enrichment analysis showed that cell proliferation in the blue modules, the apoptotic process in the turquoise module, and the cancer pathway in both modules were mostly significantly correlated with the development of CRSwNP. The noncoding RNAs (long noncoding RNA and microRNA) and the top 10 core genes in each module were found to be associated with the pathogenesis of CRSwNP. A total of nine hub genes were identified to be related to the CRSwNP phenotype. By qRT-PCR analysis, *AKT1, CDH1, PIK3R1, CBL, LRP1, MALAT1,* and *XIST* were proven to be associated with the pathogenesis of CRSwNP. *AGR2, FAM3D, PIP, DSE,* and *TMC* were identified to be related to the CRSwNP phenotype. Further exploration of these genes will reveal more important information about the mechanisms of CRSwNP.

## 1. Introduction

Chronic rhinosinusitis (CRS) is highly prevalent, affecting approximately 11% to 15% of the adult population [1, 2] and contributing to annual direct healthcare costs of $11 billion [3]. CRS is a heterogeneous group of diseases with common symptoms and clinical findings, but different pathophysiologies. In the literature, CRS has been divided into types based on the presence (CRSwNP) or absence (CRSsNP) of nasal polyps (NPs) [4]. CRSwNP is a chronic inflammatory disease that is characterized by inflammation of the nasal mucosa, nasal obstruction, and the growth of CRSwNP [3]. Because CRSwNP is prone to relapse and brings great pain to patients, it is particularly urgent to understand its molecular mechanism, as well as to promote research on related drugs. Previously, some genes such as *SRC* [5], *SMAD3* [6], and *CDH1* [7] have been found to play roles in the pathogenesis of CRSwNP. A study showed that genetic factors might play a role in the higher prevalence of nasal polyps in Asian patients compared with patients from Western countries. Therefore, exploration of the pathogenesis of CRSwNP from the perspective of genes is required.

With the development of microarray and high-throughput sequencing technology, various databases have accumulated large amounts of systematic genetic information. This has laid the foundation for us to systematically study the biological processes of diseases by constructing gene networks. Weighted gene coexpression network analysis (WGCNA) is a systematic biological method that is employed to explore the complicated relationship between genes and phenotypes among different samples. The unique advantage of WGCNA is that it can transform gene expression data into coexpression modules, providing phenotypic characteristics of interest. It can be used to identify candidate biomarker genes or therapeutic targets. WGCNA has been used to compare differentially expressed genes (DEG) and to help explore genetic interactions among different modules. It has been reported that WGCNA is successfully applied in a variety of diseases, such as subchondral bone in osteoarthritis [8], spinal cord injury [9], Wilms' tumor [10], and uveal melanoma [11]. However, WGCNA has not been applied to the analysis of the gene coexpression relationship in CRSwNP.

WGCNA identifies potential interactions and correlations between genes by determining the coexpression of gene among samples. Genes in a coexpression network are considered to be connected, and each connection has its own strength. It is worth noting that genes collected in tightly connected groups in the network are considered modules, where the most closely linked genes are defined as the “hubs.” The gene modules or clusters identified by WGCNA are closely associated with the phenotypic characteristics of samples in the gene expression profile. And studying the functions and ontologies of the genes in this module can shed light on the underlying physical mechanisms related to different biological and clinical problems [8].

Therefore, in the present study, WGCNA was constructed based on data from GSE36830 and GSE107624. The former included six NP samples and 12 normal samples. The latter contained 21 NP samples and 12 normal samples. Key gene modules associated with the pathogenesis and phenotype of CRSwNP were identified, and the biological functions and pathways of genes in different modules were detected and analyzed. Hub genes in turquoise and brown modules were also revealed. We hypothesized that these genes and modules may be potential pathogenic genes or pathways of CRSwNP, which may help us understand the pathogenesis and phenotype of CRSwNP.

## 2. Materials and Methods

### 2.1. Data Information

The CRSwNP datasets GSE36830 and GSE107624 were obtained from the National Center for Biotechnology Information (NCBI) Gene Expression Omnibus (GEO) (https://www.ncbi.nlm.nih.gov/geo/). GSE36830 includes 6 NP samples and 12 normal control samples, and the platform is the [HG-U133_Plus_2] Affymetrix Human Genome U133 Plus 2.0 Array. The normal control samples included uncinate tissues from 6 subjects with CRSsNP and 6 subjects with CRSwNP and NP tissues were collected from 6 subjects with CRSwNP. GSE107624 consists of 21 NP samples and 21 normal control samples, and its platform is the [HG-U219] Affymetrix Human Genome U219 Array. The samples were isolated from NPs or control nasal mucosa and cultured and differentiated at the air-liquid interface (ALI) cell culture system. The information in both datasets is summarized in Table 1. The original data were processed using R (version 3.5.1) packets affy_1.62.0 and annotated to form an expression matrix, and the probe was matched to its gene symbols. Both datasets were analyzed separately. We screened differentially expressed genes in two sets of sample datasets, and screened genes were differentially expressed genes in disease and normal samples between two sets of sample datasets. There is no difference in batch effect.

### 2.2. Methods

#### 2.2.1. Screening DEGs

Differentially expressed genes were screened in the mRNA expression spectra of two sets of nasal polyps and normal samples (GSE36830 and GSE107624) with the R package limma_3.40.2.

#### 2.2.2. CRSwNP Potentially Related Genes

Two sets of differentially expressed genes from the screened NP expression profile data were compared with the nasal polyp-related genes included in the public database (NCBI-gene and OMIM). Additionally, the union genes were set as the potential CRSwNP-related genes.

#### 2.2.3. WGCNA Coexpression Analysis

The expression data of GSE107624 were selected to construct the expression profile of potentially related genes in CRSwNP. The WGCNA affy_1.62.0 package was used to mine the module and analyze the coexpression genes in the expression profile above (minModuleSize = 30, mergeCutHeight = 0.25, verbose = 3). Database for Annotation, Visualization, and Integrated Discovery (DAVID, v6.8) was applied to annotate the functions and pathways of the excavated modules and to identify functional dysfunction modules with functions and pathways.

#### 2.2.4. Prediction of Noncoding RNA (ncRNA) and Transcription Factors (TF) in CRSwNP (Pivot Analysis)

The mRNA-miRNA and miRNA-lncRNA interaction data were downloaded from the StarBase database (v2.0) (http://starbase.sysu.edu.cn/starbase2/). Based on the 193388 pairs of human ncRNA-mRNA included in the StarBase database, the pivot nodes [ncRNA(miRNA, lncRNA)] of the regulatory dysfunction module were searched. To account for the 9396 pairs of human TF-mRNA recorded in the TRRUST v2 database (http://www.grnpedia.org/trrust/), the pivot nodes (TF) of the regulatory dysfunction module were searched.

#### 2.2.5. Establishment of a Multifactor (ncRNA and TF) Regulatory Network for CRSwNP

Using the information collected on the regulatory relationship between the pivot (ncRNA and TF) and the CRSwNP module using the method described above, Cytoscape (v3.4.0) (http://www.cytoscape.org/) was applied to construct the nasal polyp multifactor (ncRNA and TF) regulatory network.

#### 2.2.6. Screening of Differentially Expressed miRNA

Differentially expressed miRNA was screened from the miRNA expression profile data of CRSwNP and normal control samples (GSE107624) using the limma package. The ceRNA network of lncRNA-miRNA-mRNA was constructed according to the relationship between differentially expressed miRNA, differentially expressed genes, and lncRNA.

#### 2.2.7. Identification of Exogenous Core Genes

The protein-protein interaction data (PPI) were downloaded from the Human Protein Reference Database (HPRD) (http://www.hprd.org/), and each module above was input into the PPI. The protein interaction subnet was constructed, and the connectivity was analyzed. The genes with high connectivity were identified as exogenous core genes (drive factors).

#### 2.2.8. Coexpression of Key Genes Mediates NP Phenotype

The key coexpression genes were further used to classify CRSwNP. According to the method of finding the best sum of the squared error (SSE) inflection point to determine the optimal *K* value, the unsupervised clustering method *K*-means combined with t-distributed stochastic neighbor embedding (t-SNE) dimension reduction was used to classify CRSwNP into different phenotypes. The expression patterns of these coexpressed key genes in different subclasses were examined, and the genes with significant differences in the expression of different subclasses were analyzed (*t*-test, *P* < 0.05). These genes may be potential markers of CRSwNP subclass.

### 2.3. Experimental Validation by Quantitative Real-Time Reverse Transcriptase Polymerase Chain Reaction (qRT-PCR) Analysis

The few genes (listed in Table 2) with the most connectivity in each module were selected for experimental validation by qRT-PCR analysis. Our study included 24 NP samples and 24 normal samples, all of which were stabilized in RNAlater solution (Invitrogen, Vilnius, Lithuania). Participants in the study signed informed consent forms prior to participation in the study. The Shandong Provincial Hospital Ethics Committee approved this research. The 24 NP tissues were harvested from 20 male and 4 female patients, with a mean age of 43.22 ± 8.13. The 24 normal samples (healthy inferior turbinate tissue) were distributed in 17 males and 7 females, with a mean age of 46.06 ± 7.51. All of them underwent functional endoscopic sinus surgery or inferior turbinoplasty. All the CRSwNP subjects met the entry criteria for the CRSwNP European and American guidelines. The patients with CRSwNP ever had an unsuccessful medical therapy (oral and/or glucocorticoids, antibiotics, and antihistamines for >12 weeks). In the control group, none of the patients had taken antibiotics, antihistamines, or glucocorticoids for 4 weeks before the study. And no one had asthma, aspirin intolerance, or allergic rhinitis. The demographic characteristics of all subjects enrolled in this study are listed in Table 3. Tissue RNA preserved in RNAlater solution was isolated with prepared RNA-Quick Purification Kits (Yishan, Shanghai, China), in accordance with the manufacturer's recommendations. cDNA was synthesized using PrimeScript RT (Takara, Shiga, Japan). All qPCRs were performed in duplicate. The qRT-PCR analysis was performed with the Roche LightCycler 480 II System. GAPDH gene expression was used as an endogenous control for normalization. The relative gene expression was calculated using standard ΔΔCt methods with Roche LightCycler 480 software. A set of primers and probes was designed and optimized for these genes. The primers used in qRT-PCR are shown in Table 2.

## 3. Results

### 3.1. Screening of DEGs

**Figure 1:**
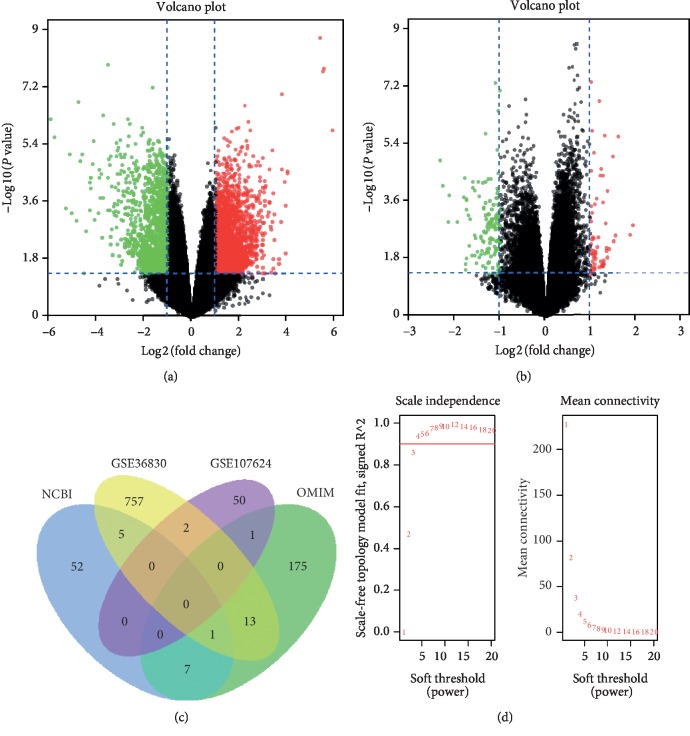
Volcano plot of differentially expressed genes (DEGs) in GSE36830 (a) and GSE107624 (b). The red point in the plot represents upregulated RNAs and the blue point represents downregulated RNAs with statistical significance. (c) A total of 1063 potential genes of chronic rhinosinusitis with nasal polyps (CRSwNP) were obtained. (d) Analysis of the scale-free fit index and mean connectivity for various soft threshold powers. Three was the optimal value.

To build the gene coexpression networks, the raw data of GSE36830 and GSE107624 were downloaded from the GEO database. R package annotations were constructed to match probes and gene symbols, and probes that matched multiple genes were removed. A total of 778 differentially expressed genes (Figure 1(a)) were screened in GSE107624, and 54 DEGs (Figure 1(b)) were screened in GSE36830. |log 2 fold change| > 2 and a false discovery rate (FDR) adjusted to *P* < 0.05 were considered DEGs. Then, the two groups of DEGs were compared with the NP-related genes in the public database (NCBI and OMIM), and a total of 1063 potential genes (Figure 1(c)) of CRSwNP were obtained.

### 3.2. Construction of the Weighted Coexpression Network and Identification of Key Modules

**Figure 2:**
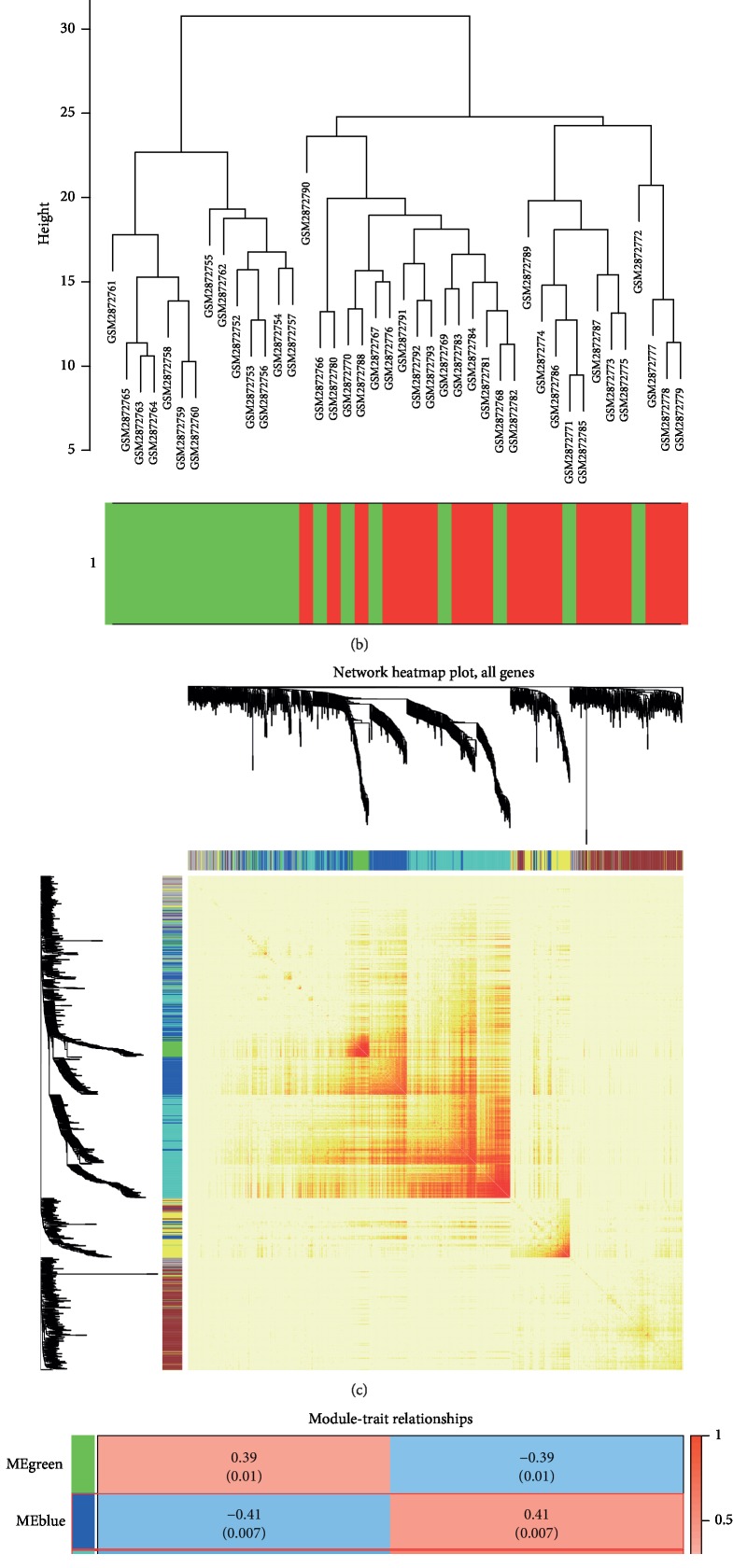
Construction of coexpression modules by weighted gene coexpression network analysis (WGCNA) in R. (a) Module clustering dendrogram. Each branch in the figure represents one gene, and every color below represents one coexpression module. (b) Sample clustering dendrogram. (c) Coexpression clustering heatmap. The different colors of the horizontal axis and vertical axis represent different modules. The yellow area in the center of the image represents the connectivity of the different modules. (d) Six modules were generated. The modules shown in the blue and turquoise modules were more strongly correlated with CRSwNP (red frame).

WGCNA analysis was performed using the expression profiles of 1,063 potentially relevant genes in the obtained nasal polyps. We analyzed the soft threshold power of the network topology with threshold weights from 1 to 20 and determined the scale independence and mean connectivity of WGCNA. An optimal threshold of 3 was selected to produce a hierarchical clustering tree of 1,063 genes (Figure 1(d)). Finally, a module clustering dendrogram (Figure 2(a)), a sample clustering dendrogram (Figure 2(b)), and a coexpression clustering heatmap (Figure 2(c)) were constructed. Six modules were generated and are shown in Figure 2(d). Blue (0.41, *P*=0.0007) and turquoise modules (0.59, *P*=4*e* − 5) were more strongly correlated with CRSwNP, while the green module (0.39, *P*=0.01) was more strongly correlated with the normal control. In the control and disease columns, positive values indicated a correlation, and *P* < 0.05 indicated statistically significant.

### 3.3. CRSwNP Functional and Pathway Enrichment Analysis in the Blue and Turquoise Modules

**Figure 3:**
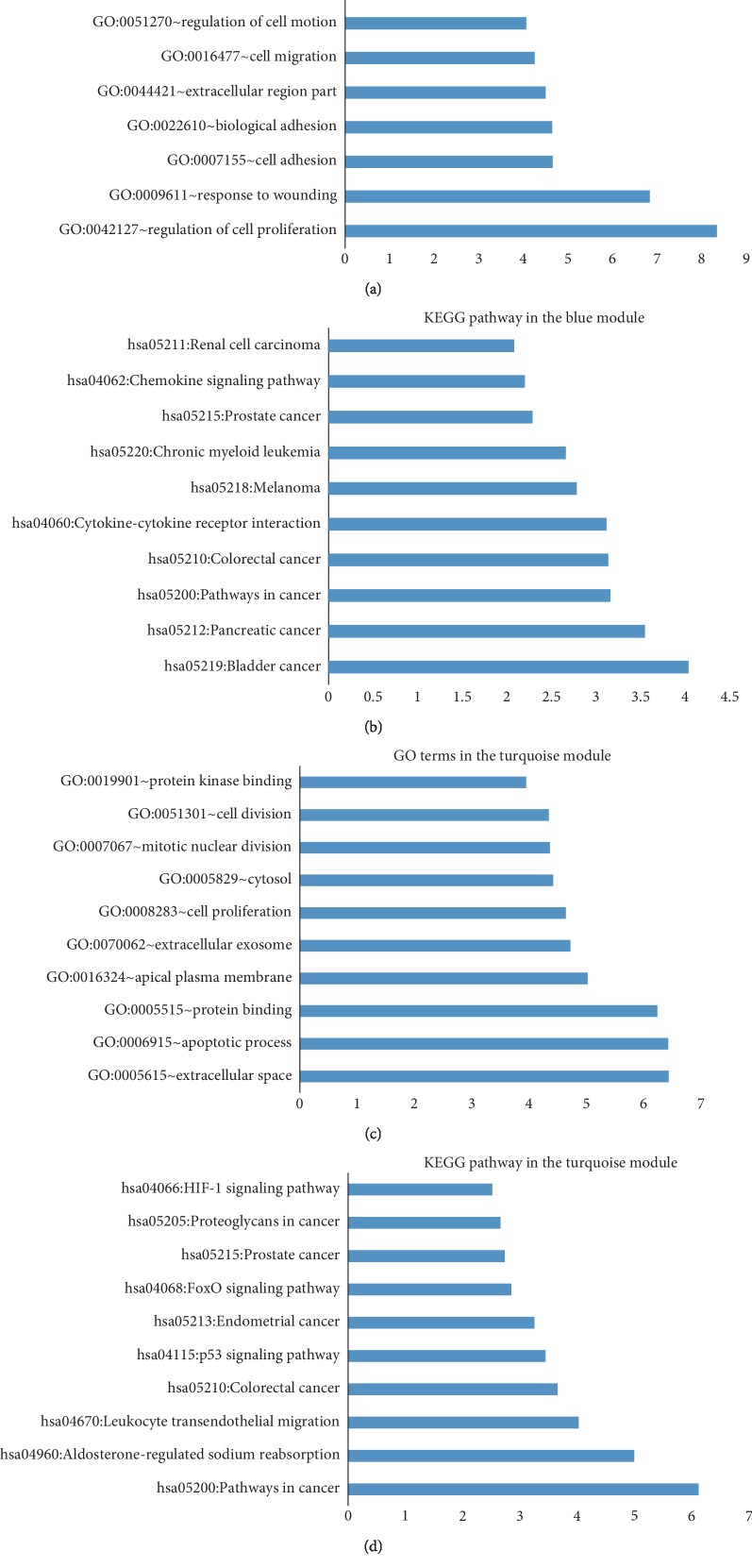
Gene Ontology (GO) terms and Kyoto Encyclopedia of Genes and Genomes (KEGG) pathways identified in the blue (a, b) and turquoise (c, d) modules. The regulation of cell proliferation and apoptotic process were the main biological processes identified in the blue and turquoise modules, respectively. The majority of pathways identified in both modules were cancer pathways.

Gene Ontology (GO) enrichment and Kyoto Encyclopedia of Genes and Genomes (KEGG) enrichment analyses were performed on the genes in the CRSwNP module using DAVID. As the results show, the blue module incorporated functions from GO, including the regulation of cell proliferation and responses to wounding, cell adhesion, and biological adhesion (Figure 3(a)). The blue module was enriched in pathways, including those associated with cancer and cytokine-cytokine receptor interactions (Figure 3(b)). In the turquoise module, the results of GO enrichment analysis mainly identified genes involved in the extracellular space, apoptotic processes, protein binding, and the apical plasma membrane (Figure 3(c)). In the KEGG pathway analysis, the identified pathways were associated with cancer, aldosterone-regulated sodium reabsorption, leukocyte transendothelial migration, colorectal cancer, and HIF-1, FoxO, and the p53 signaling pathway (Figure 3(d)).

### 3.4. Construction of CRSwNP Multifactor (ncRNA and TF) Regulation Network

**Figure 4:**
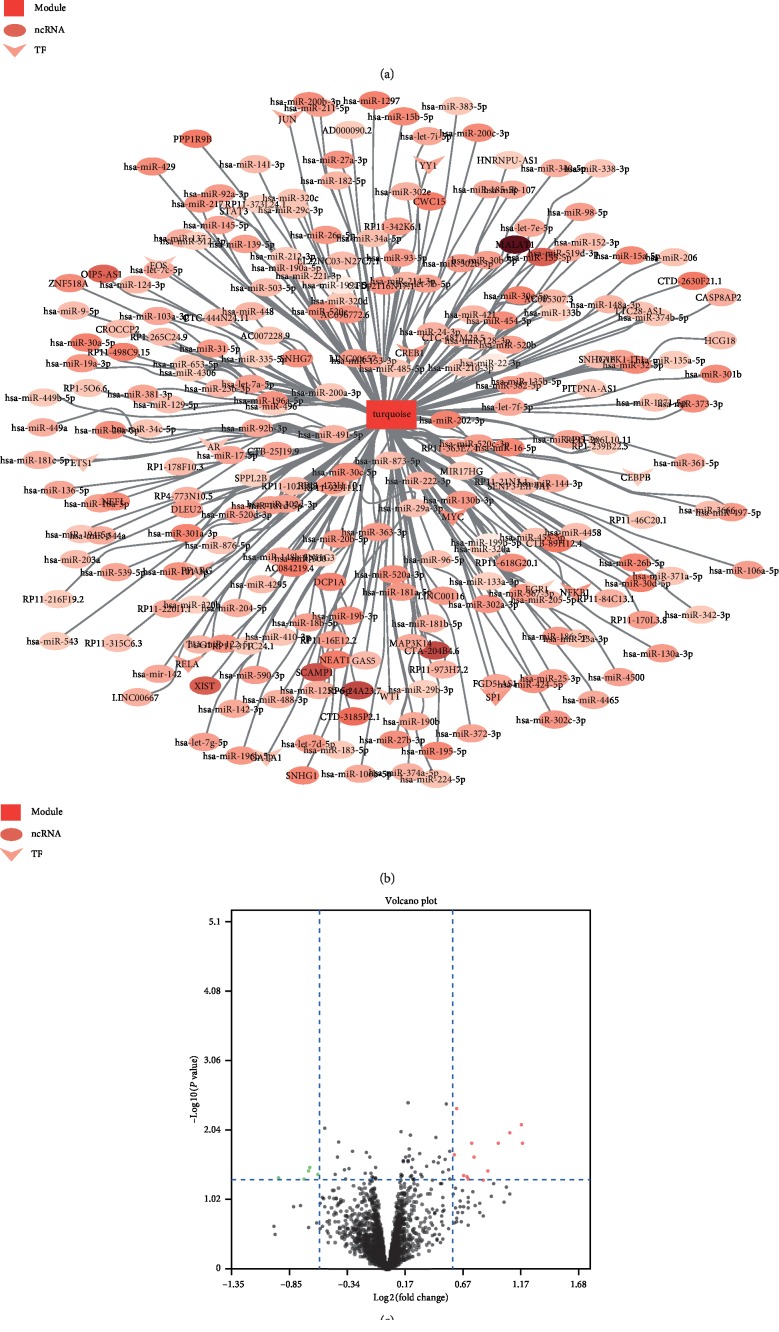
Construction of CRSwNP multifactor ncRNA and transcription factors (TF) regulation network in the blue (a) and turquoise (b) modules. The circle and V type represent ncRNA and TF, respectively. The darker the color is, the more significant the relationship between the pivot and the module is. (c) The volcano map of 18 differentially expressed ncRNAs. (d) The ceRNA network with ncRNA-miRNA-mRNA. Rectangles, ovals, and prisms represent DEGs, ncRNA, and DE_miRNA, respectively.

Using the 193388 ncRNA-mRNA interaction relationship included in the StarBase database as the background of interaction, pivot nodes (ncRNA) regulating the CRSwNP module (blue and turquoise modules) were searched (*P* < 0.05, connection > 2). The first 10 pivot node points with the most significant *P* value were selected for each module. The pivot (TF) node (*P* < 0.05, connection > 2) for regulating the CRSwNP function module (blue and turquoise modules) was searched against the background of the regulatory relationship on 9396 pairs of human TF-mRNA in the TRRUST v2 database. The blue and turquoise modules and their regulatory factors were visualized, as shown in Figures 4(a) and 4(b). The circle represented ncRNA, and the V type represents the transcription factor. The darker the color is, the more significant the relationship between the pivot and the module is.

### 3.5. Screening of Differentially Expressed miRNAs and Construction of ceRNA Networks

The limma package was used to screen the differentially expressed miRNAs in GSE107624. |log 2 fold change| > 2° or |fold change| < 2/3 and *P* < 0.05 were set as the thresholds. A total of 18 differentially expressed ncRNAs were screened, as shown in the volcano map (Figure 4(c)). From the data on the miRNA-ncRNA and miRNA-mRNA interaction in the StarBase database, we obtained 18 different lncRNA-miRNA and miRNA-mRNA relationship pairs that were different from each other. If these lncRNAs were identified as pivot nodes and the mRNA was a DEG, the lncRNA-miRNA-mRNA would form a ceRNA network, as shown in Figure 4(d). In the blue and turquoise modules, the most significantly differentially expressed ncRNAs were lncRNAs, which are listed in Table 4.

### 3.6. Identification of Exogenous Core Genes

**Figure 5:**
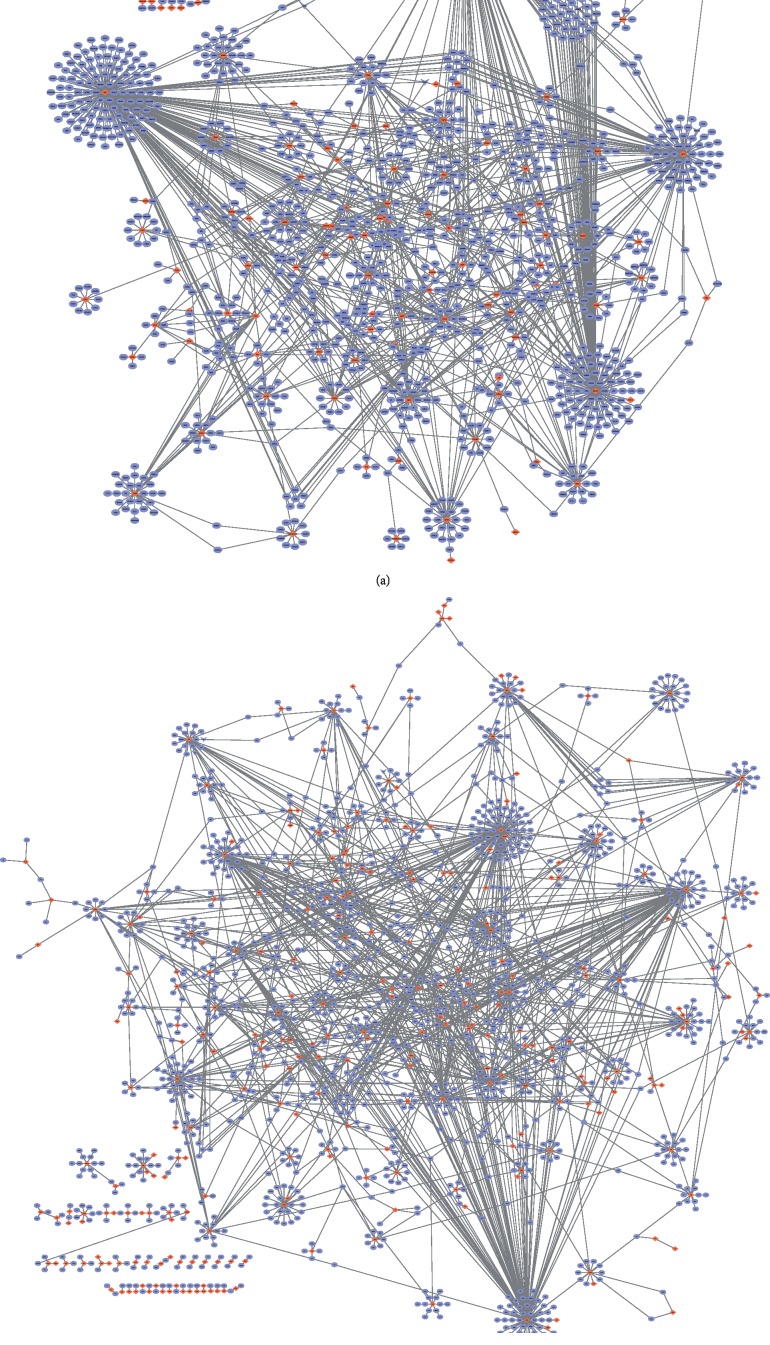
Identification of exogenous core genes and proteins interactions in the blue (a) and turquoise (b) modules. The genes with high connectivity were identified as core drive genes (drive factors). The 10 top genes were selected as core genes, as shown in Table 5.

Genes from the blue and turquoise CRSwNP modules were put into the PPI, as shown in Figures 5(a) and 5(b). Red represents the module genes, and purple represents the remaining genes in the protein interaction network. Then, the connectivity of each protein interaction subnet was analyzed, and the genes with high connectivity were identified as core drive genes (drive factors). The top 10 genes in each of the blue and turquoise modules were selected, as shown in Table 5.

### 3.7. Identification of Genes about the CRSwNP Phenotype

**Figure 6:**
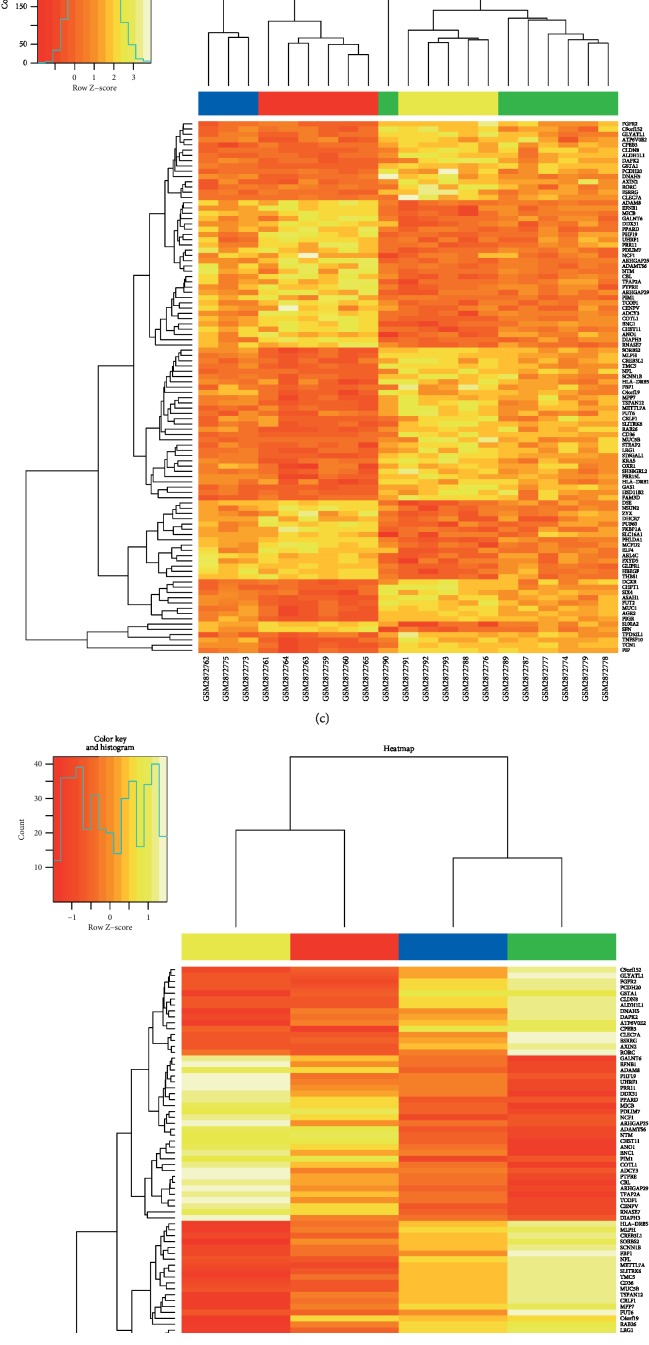
(a) Identification of the best inflection point based on the sum of the squared error (SSE). *K* = 4 is the optimal value. (b) The clustering of chronic rhinosinusitis with nasal polyps (CRSwNP) samples. It was possible to divide all the CRSwNP samples into four phenotypes, represented here by different colors. (c) Expression heatmap of genes coexpressed in CRSwNP. (d) Expression heatmap of coexpressed hub genes in the four subtypes of CRSwNP.

Based on the analysis results of the WGCNA coexpression module, the coexpression network of the blue and turquoise modules was further analyzed. The screened *N* = 101 coexpression key genes (|cor| > 0.6, *P* < 0.05) were mapped to GSE107624 for unsupervised clustering, and the CRSwNP sample (*N* = 21) was selected. The *K*-means unsupervised clustering method was used to classify all CRSwNP samples. First, the optimal value of *K* was selected by finding the inflection point of the SSE. As can be seen in Figure 6(a), the decline slows down after *K* = 4, so *K* = 4 was selected. The R package R-Tsne was used to reduce the dimensionality of the gene expression data. As shown in Figure 6(b), it was possible to divide all the CRSwNP samples into four phenotypes. The coexpression key genes in all the CRSwNP samples are shown in Figure 6(c), and the coexpression key genes in the four clusters are shown in Figure 6(d). Among them, *ST6GAL1, AGR2, FAM3D, PIP, COTL1, PHLDA1, MLPH, DSE,* and *TMC5* were differentially expressed in four clusters. Therefore, these nine genes may be related to the CRSwNP phenotype.

### 3.8. Experimental Validation

**Figure 7:**
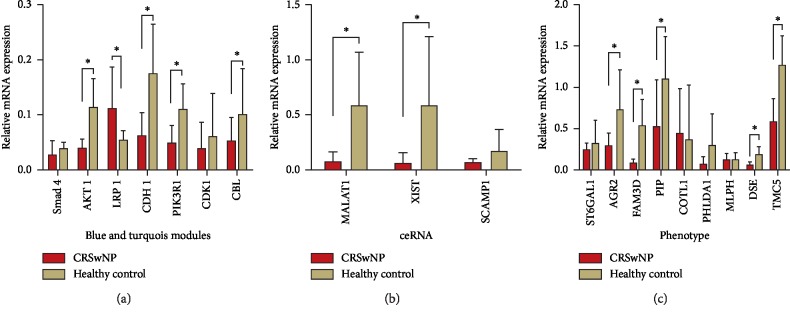
The mRNA expression in individuals with chronic rhinosinusitis with nasal polyps (CRSwNP) (*n* = 24) and healthy controls (*n* = 24) was assessed by polymerase chain reaction (qRT-PCR) analysis. Fold change was calculated relative to healthy controls. (a) mRNA expression in the blue module, turquoise module, and ceRNA. (b) mRNA expression of the CRSwNP phenotype. All data are displayed as the mean ± SD. ^*∗*^*P* < 0.05 vs. healthy control (Student's *t*-test). ncRNA, noncoding RNA; TF, transcript factor; DEG, differentially expressed genes; DE_miRNA, differentially expressed microRNA.

Based on the bioinformatics analysis results described above, we experimentally validated six genes in the blue module, three genes in the turquoise module, three lncRNAs in the ceRNA network, and all nine genes related to the CRSwNP phenotype. Although *SRC* and *SMAD3* are the first two core genes in the blue module, and they have been reported to be associated with the pathogenicity of CRSwNP in the previous literature, we decided not to include them in the experimental validation. In the turquoise module and ceRNA network, the genes with the most connectivity were selected for the experiment. In the blue and turquoise modules, the mRNA expression of *AKT1* (0.35-fold), *CDH1* (0.36-fold), *PIK3R1* (0.44-fold), and *CBL* (0.52-fold) decreased, and that of *LRP1* (2.06-fold, *n* = 24) increased, all of which were significantly different between the CRSwNP and healthy control groups. *Smad4* (0.69-fold) and *CDK1* (0.65-fold) did not show any significant difference (Figure 7(a)). In the ceRNA network, the expression of *MALAT1* (0.12-fold) and *XIST* (0.10-fold) decreased and showed significant differences between groups, while *SCAMP1* (0.12-fold) was not significantly different between groups (Figure 7(b)). The mRNA expression of the CRSwNP phenotype is shown in Figure 7(c). *AGR2* (0.39-fold), *FAM3D* (0.15-fold), *PIP* (0.46-fold), *DSE* (0.33-fold), and *TMC* (0.46-fold) decreased, and all of them were significantly different between groups, which were associated with the phenotype of CRSwNP, while *ST6GAL1* (0.77-fold), *COTL1* (1.19-fold), *PHLDA1* (0.58-fold), and *MLPH* (0.96-fold) were not significantly different between groups.

## 4. Discussion

CRSwNP, which leads to chronic inflammation of the nasal mucosa, nasal obstruction, and growth of NPs, causes a serious psychological burden and economic pressure on affected patients because of its refractory and relapse characteristics. Currently, little is known about the pathogenesis and phenotype of CRSwNP on a genetic level. In the current study, we used WGCNA to identify key modules and hub genes involved in the pathogenesis and phenotype of CRSwNP by R. The two most relevant modules of CRSwNP were identified, namely, the blue and turquoise modules, which were both positively shown to be correlated with the disease. By functional enrichment analysis, cell proliferation and apoptotic processes were found to be the most common biological processes in the blue and turquoise modules, respectively. The ceRNA network was constructed, and 10 hub genes were identified in each module as being involved in the pathogenesis of CRSwNP. Nine genes (*ST6GAL1, AGR2, FAM3D, PIP, COTL1, PHLDA1, MLPH, DSE,* and *TMC5*) were determined to be related to the phenotype of CRSwNP. By experimental validation, *AKT1, CDH1, PIK3R1, CBL,* and *LRP1* in the blue and turquoise modules and *MALAT1* and *XIST* in the ceRNA network were shown to be associated with NPs. Five genes, *AGR2, FAM3D, PIP, DSE,* and *TMC*, were identified to be related as being related to the CRSwNP phenotype.

In this work, a total of six modules (blue, brown, green, grey, turquoise, and yellow) were mined by WGCNA, and the blue and turquoise modules were shown to be most significantly correlated with CRSwNP. Using conventional experimental methods, previous studies have reported that cell proliferation plays an important role in the pathogenesis of CRSwNP [12, 13]. Through various experimental methods, other studies have also shown that nasal mucosa epithelium apoptosis is regulated by multiple molecules, which affects NP formation [14, 15]. However, in the current study, cell proliferation was identified in the GO terms of both modules, and the apoptotic processes were shown to be the most significant biological processes in the turquoise module. This suggests that the precise biological process involved in the pathogenesis of CRSwNP could be predicted by WGCNA. Future studies on the mechanisms of CRSwNP should further explore this direction.

Interestingly, in our study, both the blue and turquoise modules in the KECG pathway were remarkably enriched in the pathways associated with cancer. Previously, only one paper reported nasal polyposis is associated with malignancy. Specifically, Pourang et al found that patients with prevalent nasal polyposis are at an increased risk for malignancies of the head and neck, specifically squamous cell carcinoma, compared to individuals without nasal polyposis [16]. Moreover, TGF-*β* [17], Wnt [18], and Myc [19] pathways have been reported to be associated with CRSsNP and different cancers. This indicates that CRSwNP shares some common pathways with cancer. However, our results not only confirmed this, but also found more cancer pathways, thereby expanding our understanding of the CRSwNP mechanism. Therefore, by suppressing the growth of cancer pathways, it may also be possible to inhibit the development of nasal polyps. Additionally, the HIF-1 and p53 signaling pathways have been reported to mediate the pathogenesis of CRSwNP [20, 21]. These results support the idea that WGCNA could be used to explore useful information about the mechanisms of illness.

At present, only a few articles have reported the relationship between ncRNA and CRSwNP, mainly focusing on miRNA [17, 22], while lncRNA has not been addressed. ceRNA has important biological significance in many diseases, including cancer [23] and noncancer diseases [24]. However, little data on CRSwNP have been reported. Our study constructed the ceRNA network of CRSwNP and identified several lncRNAs and miRNAs that may be associated with the pathogenesis of CRSwNP. lncRNA *MALAT1* and *XIST* were most obviously related to CRSwNP in both modules. Although they have been reported to be correlated with cancer, they have not previously been associated with CRSwNP. By RT-PCR analysis of the top three lncRNA in the ceRNA network, *MALAT1* and *XIST* are significantly downregulated in CRSwNP, and statistically significant differences between CRSwNP patients and healthy controls were shown. These two lncRNAs have been widely reported to be related to the occurrence of cancer. This study provides further evidence that CRSwNP shares certain pathways with cancer development [25]. Additionally, based on limited experimental conditions, the miRNAs and some other lncRNA in this study have not been analyzed in experiments.

In both modules, we listed the top 10 core genes with the pathogenesis of CRSwNP. In the blue modules, *SRC, Smad3, Smad4, AKT1, LRP1, IGF1R, FGFR3, CDH1, NCF1,* and *ARRB2* were most significantly related to CRSwNP. Except for *SRC* [5], *Smad3* [6], and *CDH1* [7], no other genes were found to play a role in the pathogenesis of CRSwNP. In the turquoise module, which included *PIK3R1, CDK1, CBL, INSR, VAV1, MDM2, HCK, SOS1, PRKCE,* and *ITGB2*, no genes have been shown to be involved in the development of CRSwNP in the currently published literature studies. However, in this study, *AKT1, CDH1, PIK3R1, CBL,* and *LRP1* exhibited statistically significant differences in CRSwNP patients, compared to the healthy controls in both modules. AKT1 plays an important role in cell survival and apoptosis. PI3K-Akt signaling pathway is a classical signal pathway, which involves a variety of cancers and inflammation. It has been reported that AKT1 was associated with inflammation of liver disease and acute pancreatitis [26, 27]. Therefore, it may also play a role in rhinosinusitis, which is also an inflammatory disease. We further demonstrated that *CDH1* is significantly downregulated in CRSwNP, which is consistent with previous research results [28]. PIK3R1 (phosphoinositide-3-kinase regulatory subunit 1) is a potential therapeutic target in glioblastoma multiforme and that it also influences tumor cell growth and motility. CBL is an E3 ubiquitin-protein ligase involved in cell signaling and protein ubiquity. LRP1 is a key signaling protein and involved in various diseases, such as neurodegenerative diseases, atherosclerosis, and cancer. Most of these genes were not reported in CRSwNP. This shows that WGCNA is useful for revealing new pathogenic genes and can provide abundant reference resources for future experimental research. The potential importance of these genes will be further investigated by appropriate experiments.

We determined nine genes related to the CRSwNP phenotype. In clinical practice, CRSwNP is commonly divided into eosinophilic and noneosinophilic NP according to the presence or absence of eosinophils. However, at a genetic level, the CRSwNP phenotype has not been described at all. In this study, CRSwNP was classified into four phenotypes, and a total of nine genes were found to be associated with its occurrence. This may be a new discovery. In other words, at a genetic level, CRSwNP could be further compartmentalized. *ST6GAL1, AGR2, FAM3D, PIP, COTL1, PHLDA1, MLPH, DSE,* and *TMC*5 were differentially expressed in the four clusters. However, none of them have been studied in relation to CRSwNP. In this study, by RT-PCR analysis, five genes (*AGR2, FAM3D, PIP, DSE*, and *TMC*) were identified as being related to the CRSwNP phenotype. Interestingly, AGR2 has been found to play an important role in prostate tumorigenesis and metastasis-related phenotypes [29]. DSE was associated with musculocontractural phenotypic variability [30]. Future research will focus on experiments to explore the potential value of these genes and verify which genes belong to which phenotype of CRSwNP.

Our research shows that a large number of related genes can be mined through WGCNA, and through experimental verification, it was found that the predictions of WGCNA are mostly correct. This suggests that WGCNA is a good tool for the early exploration of disease genes. Although our method found some crucial genes and modules, there is one limitation and shortcoming in our study, which is that the datasets that we could obtain were very limited. CRSwNP datasets on GEO, especially large sample datasets, are very few in number. The results would be better if there was a larger dataset including nasal polyps and normal controls.

In summary, we identified crucial modules, biological processes, pathways, ncRNA, and hub genes related to CRSwNP. *AKT1, CDH1, PIK3R1, CBL, LRP1, MALAT1,* and *XIST* were proven to be associated with the pathogenesis of CRSwNP. *AGR2, FAM3D, PIP, DSE,* and *TMC* were identified to be related to the CRSwNP phenotype. Further exploration of these genes will reveal more important information about the mechanisms of CRSwNP.

## Figures and Tables

**Table 1:** The sample information summary.

Data	Data type	Platform	Disease vs. normal
GSE36830	RNA-seq	[HG-U133_Plus_2] Affymetrix Human Genome U133 plus 2.0 Array	6 vs. 12
GSE107624	RNA-seq	[HG-U219] Affymetrix Human Genome U219 Array	21 vs. 21
GSE107624	miRNA-seq	[miRNA-4] Affymetrix Multispecies miRNA-4 Array	21 vs. 21

**Table 2:** The genes and primers used for experimental validation by polymerase chain reaction (qRT-PCR) analysis.

Gene	Module	Primer (*F*)	Primer (*R*)
*Smad4*	Blue	CTGGAGGTGGCCTGATCTTC	ACGATGGCTGTCCCTCAAAG
*AKT1*	Blue	GGACAAGGACGGGCACATTA	CGACCGCACATCATCTCGTA
*LRP1*	Blue	CTGGCGAACAAACACACTGG	CACGGTCCGGTTGTAGTTGA
*CDH1*	Blue	GGGGTCTGTCATGGAAGGTG	GAAACTCTCTCGGTCCAGCC
*PIK3R1*	Turquoise	GCTTTGCCGAGCCCTATAAC	GAGCCCTTTGCTTTCCAGAG
*CDK1*	Turquoise	ACTACAGGTCAAGTGGTAGCC	TCCATGTACTGACCAGGAGG
*CBL*	Turquoise	TGTTGGAGCAGAATCCCGAC	GATCACTGGAACTTGGGGCA
*MALAT1*	ceRNAs	GGATTCCAGGAAGGAGCGAG	ATTGCCGACCTCACGGATTT
*XIST*	ceRNAs	TTCTAGTCCCCCAACACCCT	TGGAGGACGTGTCAAGAAGAC
*SCAMP1*	ceRNAs	GCCGCAGAATTAGATCGTCG	TGAACTGTCACTCACATCCAC
*ST6GAL1*	Phenotype	GAGTTCCTCCCATCCAAGCG	TCATCTGTGCCCTGGTTGAG
*AGR2*	Phenotype	ACACAAAGGACTCTCGACCC	GGACAAACTGCTCTGCCAAT
*FAM3D*	Phenotype	GACTTGGGGAGTTCCTACGC	TACCCCTGAGGTCTTTGGCT
*PIP*	Phenotype	GCTCAGGACAACACTCGGAA	TTGTCGTCACATAGGCAGGC
*COTL1*	Phenotype	GCACACACGTCCATTCCCTA	TTCTCACCACCGAGCAATCC
*PHLDA1*	Phenotype	GAGGAAGGGCTGCTGCTTAT	GCAGTTCCTTGAGCTTGACC
*MLPH*	Phenotype	TCAACGAGATTTTGACCTCCG	GTGGGTCTCGTTCAGATGGG
*DSE*	Phenotype	TTGTGGATGCTGTCCCTGAT	GTAGTTGTCACCATCCGTGC
*TMC5*	Phenotype	CAGTTCACTGGGCTGGAGTT	ATGTAGGCCAGCTGCATGTT

**Table 3:** The demographic characteristics of all subjects.

	Control	CRSwNP
No. of patients	24	24
Sex, male/female	17/7	20/4
Age (y)	46.06 ± 7.51	43.22 ± 8.13
Duration (y)	0	2.3 (1.8–4.5)
Asthma history, yes/no	0/24	0/24

**Table 4:** The differentially expressed noncoding RNAs (ncRNAs) with the highest significant (*P* values) in the blue and turquoise modules.

Module	ncRNA-pivot	*P*	Connection
Blue	MALAT1	4.76 × 10^10^	33
Blue	XIST	1.40 × 10^9^	61
Blue	SCAMP1	3.50 × 10^9^	11
Blue	OIP5-AS1	3.50 × 10^9^	11
Blue	NEFL	1.13 × 10^7^	5
Blue	CTA-204B4.6	1.80 × 10^7^	14
Turquoise	XIST	4.20 × 10^7^	93
Turquoise	MALAT1	4.07 × 10^10^	48

**Table 5:** The 10 hub genes in the blue and turquoise modules.

Module	TF-pivot	Degree
Blue	SRC	207
Blue	SMAD3	180
Blue	SMAD4	152
Blue	AKT1	117
Blue	LRP1	54
Blue	IGF1R	51
Blue	CDH1	38
Blue	CASK	37
Blue	NCF1	36
Blue	ARRB2	36
Turquoise	PIK3R1	128
Turquoise	CDK1	119
Turquoise	CBL	85
Turquoise	INSR	74
Turquoise	VAV1	62
Turquoise	MDM2	57
Turquoise	HCK	55
Turquoise	SOS1	45
Turquoise	PRKCE	45
Turquoise	ITGB2	37

Degree represents the connectivity of each gene or protein interaction.

## Data Availability

The WGCNA R data used to support the findings of this study have not been made available because the data are based on third-party analysis, and based on confidentiality, they are refused to be disclosed to the public. The RT-PCR data used to support the findings of this study are available from the corresponding author upon request.

## Abbreviation

CRS: Chronic rhinosinusitis
NPs: Nasal polyps
WGCNA: Weighted gene coexpression network analysis
NCBI: National Center for Biotechnology Information
David: Database for Annotation, Visualization, and Integrated Discovery
GO: Gene Ontology
KEGG: Kyoto Encyclopedia of Genes and Genomes
GEO: Gene Expression Omnibus
DEG: Differentially expressed genes
HPRD: Human Protein Reference Database
ncRNAs: Noncoding RNAs
lncRNA: Long noncoding RNAs
miRNA: MicroRNA.
